# Gene tree correction guided by orthology

**DOI:** 10.1186/1471-2105-14-S15-S5

**Published:** 2013-10-15

**Authors:** Manuel Lafond, Magali Semeria, Krister M Swenson, Eric Tannier, Nadia El-Mabrouk

**Affiliations:** 1 Département d'Informatique (DIRO), Université de Montréal, H3C3J7, Canada; 2 Laboratoire de Biométrie et Biologie Évolutive, UMR CNRS 5558, Université Lyon I, F-69622 Villeurbanne, France; 3 INRIA Grenoble Rhône-Alpes, F-38334 Montbonnot, France; 4 McGill Center for Bioinformatics, McGill University, H3C2B4, Canada

## Abstract

**Background:**

Reconciled gene trees yield orthology and paralogy relationships between genes. This information may however contradict other information on orthology and paralogy provided by other footprints of evolution, such as conserved synteny.

**Results:**

We explore a way to include external information on orthology in the process of gene tree construction. Given an initial gene tree and a set of orthology constraints on pairs of genes or on clades, we give polynomial-time algorithms for producing a modified gene tree satisfying the set of constraints, that is as close as possible to the original one according to the Robinson-Foulds distance. We assess the validity of the modifications we propose by computing the likelihood ratio between initial and modified trees according to sequence alignments on Ensembl trees, showing that often the two trees are statistically equivalent.

**Availability:**

Software and data available upon request to the corresponding author.

## Introduction

A gene tree represents the evolutionary relationships between a set of homologous genes. Gene trees are useful to unveil the molecular evolutionary events that have shaped today's genomes. They are traditionally constructed from sequence alignments [1], while recent methods also use the information from species phylogenies through reconciliation [2-8]. But constructing good gene trees is still challenging: for example, while they yield orthology and paralogy relationships between genes, often alternative or additional information, such as conserved synteny, is used to provide or confirm orthology [9].

The orthology information suggested by gene tree reconciliation may be contradictory with that suggested by an external source, such as conserved synteny [10,11]. We explore a way to reconcile them by performing slight modifications to a given gene tree in order to fit external information on orthology.

We propose two kinds of gene tree modification, which consist in computing a gene tree as close as possible to the initial one, satisfying two kinds of constraints. One kind is a set of pairs of genes that should be orthologous but are seen as paralogous in the initial tree. This occurs when orthologs are computed with synteny for example [11]. The other kind is a set of clades that should be rooted by speciation nodes but are rooted by duplication nodes in the initial tree. This occurs when dubious duplications are detected because of the absence of extant support for a duplication, or because of ancestral synteny information [10]. We give polynomial-time algorithms for both problems under the Robinson-Foulds distance, thus proposing several ways to improve gene trees according to external information.

There are very few gene tree reconstruction methods including synteny information [12], whereas integrating this information could be valuable [13]. The modifications we propose could be included in a local search framework as other kinds of modifications based on duplications and losses [14-17]. We assess the validity of the modifications we propose by computing the likelihood ratio between initial and modified trees according to sequence alignments on Ensembl trees [18], showing that often the two trees are statistically equivalent.

## Different gene tree corrections

### Phylogenies

A *phylogeny *is a rooted binary tree which represents the evolutionary relationships between the nodes. Internal nodes are extinct ancestors, leaves are extant elements and edges represent direct descents between parents and children. Given a node *x *of a phylogeny *T*, we call an *ancestor *of *x *any node on the path from the root (inclusively) of *T *to the parent of *x*. For a leaf-subset *X *of *T*, lca*_T _*(*X*), the *lowest common ancestor *of *X*, denotes the farthest node from the root which is an ancestor of all elements of *X*. We use the notation *l*(*x*), and call the *clade *of *x*, the set of leaves which are descendant from an internal node *x*. We also denote by *l*(*T *) the set of leaves, and by *V*(*T *) the set of nodes of *T*.

We define two kinds of phylogenies: species trees and gene trees. Species are identified with *genomes*. For our purpose, genomes are simply sets of genes. Therefore, each gene *g*, extant or ancestral, belongs to a species *s*(*g*). We then have one species tree *S*, where nodes are identified with species, and many gene trees, where nodes are identified with genes. The set of genes in a gene tree is called a *gene family*.

A *reconciliation *between a gene tree *G *and a species tree *S *consists in assigning to each gene *g *of *G *(both extant and ancestral) the species *s*(*g*) corresponding to the lowest common ancestor in *S *of the set {*s*(*l*), for all *l ∈ l*(*g*)}. Every internal node *g *of *G *is labeled by an *event E*(*g*), verifying *E*(*g*) = speciation if *s*(*g*) is different from *s*(*g*_ℓ_) and *s*(*g_r_*) where *g*_ℓ _and *g_r _*are the two children of *g*, and *E*(*g*) = duplication otherwise.

**Figure 1:**
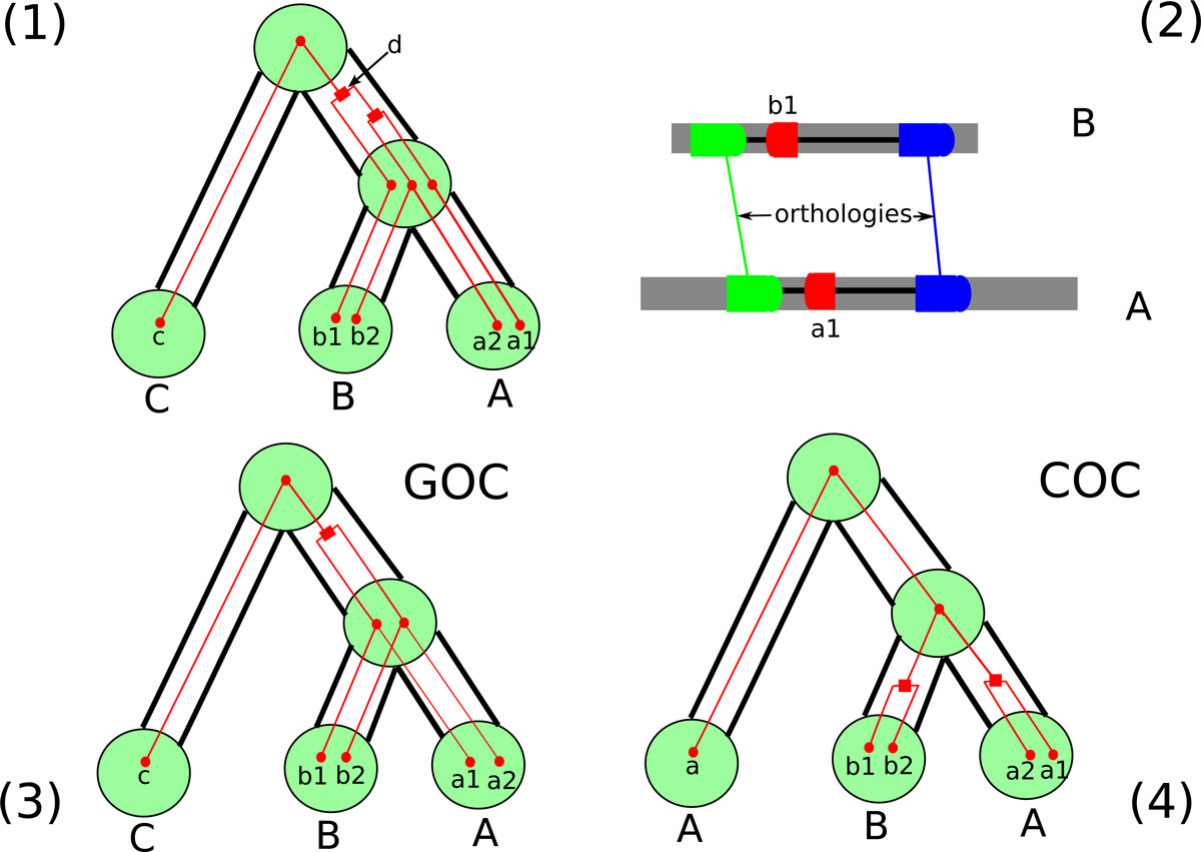
**Description of the two problems**. (1) A gene tree (the "initial tree") for the gene family {*c, b*1*, b*2*, a*1*, a*2} is shown with small red nodes and single thin red edges. It is reconciled with the phylogeny of the three species *A*, *B *and *C *shown with large green nodes and hollow edges represented by a pair of parallel black lines. Duplication nodes of the reconciled gene tree are squared, while speciation nodes and leaves are dots. (2) The two neighbors of *b*1 on genome *B *and of *a*1 on genome *A *are inferred to be orthologous according to their lowest common ancestor in their respective gene trees (not shown). This is an argument for infering orthology between *b*1 and *a*1, which is in contradiction with the information provided by the initial tree: their lowest common ancestor is a duplication, and thus they are inferred to be paralogous. (3) A solution to the GOC problem, that is a gene tree of minimum RF distance with the initial tree verifying the constraint of *b*1 and *a*1 being orthologous. (4) A solution to the COC problem, that is a reconciled tree in which the clade {*b*1*, b*2*, a*1*, a*2} of *d *in the initial tree is rather rooted by a speciation node in the corrected tree. This is an example where the optimal solutions to the two problems differ.

The reconciliation of *G *and *S *gives all informations about the gene family history. In particular it defines the gene content of an ancestral species at the time of speciation. A reconciliation also implies the orthology and paralogy relationships between genes: two genes *g *and *g*' of *T *are said to be *orthologous *if *E*(lca*_T_*(*g, g*')) = speciation; *g *and *g*' are *paralogous *if *E*(lca*_T_*(*g, g*')) = duplication. For example, Figure 1(1) shows a gene tree reconciled with a species tree. In this gene tree *a*_1 _and *b*_1 _are paralogous as their lowest common ancestor is *d *which is a duplication node, while *a*2 and *b*2 are orthologous. The number of dots inside big circles represents the number of genes in the corresponding genome (each big circle represents a species).

### The Robinson-Fould (RF) distance

The RF distance *RF *(*G, G*') between two phylogenies *G *and *G*' is the cardinality of the symmetric difference between the clade-sets of the two trees. In other words, denote by *c*(*G, G*') the number of clades that are in *G *but not in *G*'. Then *RF *(*G, G*') = *c*(*G, G*') + *c*(*G*'*, G*).

In this paper, since we only compare rooted binary trees sharing the same leaf-sets, they always have the same number of internal nodes, and hence the same number of clades. Therefore *c*(*G, G*') = *c*(*G*'*, G*), and *RF *(*G, G*') = 2*c*(*G, G*').

### Two correction problems

Suppose that in addition to a species tree and a set of reconciled gene trees, we are given additional information of two kinds:

• Pairs of genes that we know are orthologous;

• Duplication nodes of some gene trees that we suspect to be false.

Constraints of orthology on pairs of genes may for example be generated from synteny analysis [9,11]. Some pairs may contradict the information given by the gene tree. Let *P *be a set of pairs (*g*_1_*, g*_2_) of orthologous extant genes (verifying *s*(*g*_1_) ≠ *s*(*g*_2_)). A gene tree *G *is said to *satisfy *a set *P *if, for any pair (*g*_1_*, g*_2_) ∈ *P*, lca*_G_*(*g*_1_*, g*_2_) is a speciation node.

Problem 1 *Gene Orthology Correction [GOC] Problem*

***Input***: *A gene tree G reconciled with a species tree S, and a set P of gene pairs that are required to be orthologous;*

***Output***: *A corrected gene tree G_P _satisfying P, such that RF *(*G, G_P _*) *is minimum among all possible solutions*.

An example is given in Figure 1: (1) is the initial tree, and (2) depicts two syntenic regions of size 3 surrounding genes *b*1 and *a*1. In general (if we neglect the effect of gene conversion) genes in two syntenic regions should be either all pairwise orthologous or all pairwise paralogous [11]. Consequently, if the two neighbors of *b*1 on genome *B *and of *a*1 on genome *C *are inferred to be orthologous (according to their lowest common ancestor in their respective gene trees), then an orthology constraint should be imposed on the pair (*b*1*, a*1). Figure 1. This principle is usually considered as one of the most efficient method to detect orthologies [9]. (3) is a corrected tree.

On the other hand, duplication nodes of a gene tree can be considered dubious for different reasons. For example, in Ensembl [19], "dubious" is a label assigned to the non-apparent duplication nodes [20,21] pointing to an incongruence between the gene tree and the species tree. Alternatively, inferred ancestral synteny may also point to dubious duplication nodes [10]. Formally, clades corresponding to some duplication nodes may erroneously be considered as sets of paralogous genes, and should rather be considered as orthologous.

A gene tree *G *is said to *satisfy *a set *C *of its clades if *E*(lca*_G_*(*c*)) = speciation for all *c ∈ C*.

Problem 2 *Clade Orthology Correction *[*COC*] *Problem*

***Input***: *A gene tree G reconciled with a species tree S, and a set C of clades of G assigned to duplication nodes;*

***Output***: *A corrected tree GC satisfying C, such that RF *(*G, G*_*C*_) *is minimum among all possible solutions*.

The two problems are different, as exemplified by Figure 1, where (3) is an optimal solution to GOC while (4) is an optimal solution to COC, the latter more distant to the initial tree.

In the next two sections, we use *S *for the species tree name, *G *for the reconciled gene tree, and we give efficient solutions to these two problems.

## The Gene Orthology Correction Problem

Notice that for any instance of the GOC problem, a corrected tree satisfying *P *always exists. Indeed, for any extant species *x *of *S*, one can make a tree whose leaf-set is all the extant genes *g *of *G *for which *s*(*g*) = *x*. Doing this for every species yields a forest whose roots can be reconnected by matching the topology of *S*, ensuring that any pair of genes not in the same species are orthologous. However, the obtained tree can be very far from the original.

Let *P *be a set of gene pairs (which are leaves of *G*) required to be orthologous. Notice that if (*a, b*) ∈ *P*, then we also have (*b, a*) ∈ *P*. For any pair (*a, b*) ∈ *P*, if lca*_G_*(*a, b*) is a duplication in *G*, then (*a, b*) is a pair of *false paralogs*. The set *P_f _*⊆ *P *denotes the set of all false paralogous pairs of *P*.

Given two distinct leaves *a *and *b *of *G*, we set *r_a,b _*= lca*_G_*(*a, b*), *s_a,b _*= lca*_S _*(*s*(*a*)*, s*(*b*)), and define *h_a,b _*as the highest node (closest to the root) on the path from *a *to *r_a,b _*such that *s*(*h_a,b_*) is a descendant of *s_a,b_*. Notice that *h_a,b _*can be *a *itself, but not *r_a,b_*.

**Figure 2:**
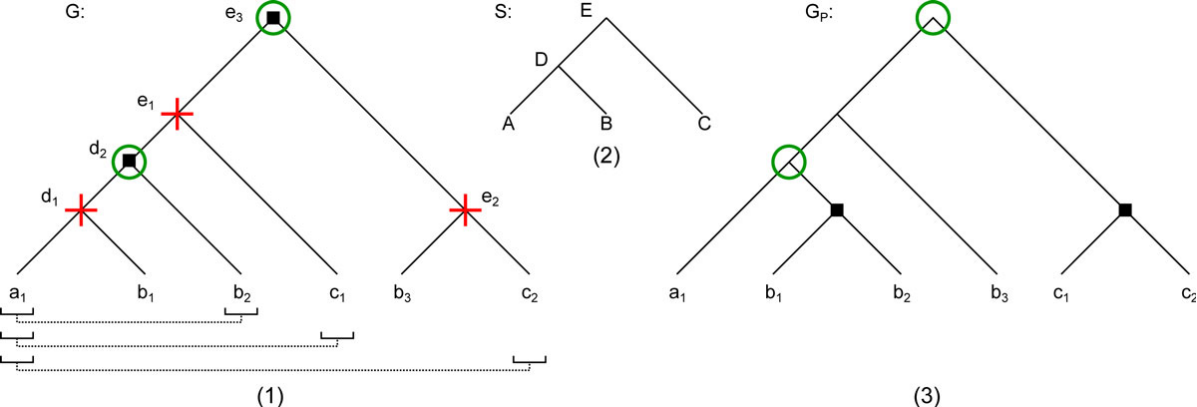
**GOC Procedure**. (1) A gene tree *G *reconciled with species tree *S*. Duplication nodes are denoted by a black square. The leaves and internal nodes of *G *are labeled with the letter of their corresponding species. Brackets denote the required orthologs given by the input set *P *= {(*a*_1_*, b*_2_), (*a*_1_*, c*_1_), (*a*_1_*, c*_2_)}. The non-preservable nodes (nodes of *H*) are depicted by red crosses, while preservable nodes are circled in green. (2) The species tree associated with *G*. (3) The tree *G_P_*, a solution to the GOC problem, which preserves every possible clade.

For instance on Figure 2(1), *a*_1_, *c*_2 _are false paralogs with $r_{a_{1},c_{2}}=e_{3}$ and $s_{a_{1},c_{2}}=E$. From this, one can deduce that $h_{a_{1},c_{2}}=d_{2}$ and $h_{c_{2},a_{1}}=c_{2}$. We show below that, for any pair (*a, b*) of false paralogs, *h_a,b _*is the highest node on the path from *a *to *r_a,b _*over which we can move *b *to make lca*_G_*(*a, b*) a speciation node. The reason for moving *b *as high as possible is to preserve as many clades as possible, allowing a minimum RF distance between the initial and corrected tree.

**Lemma 1 ***Let *(*a, b*) *be a pair of false paralogs in G, and let G*' *be a tree in which a and b are orthologous. If x is an ancestor of h_a,b _and a descendant of r_a,b_, then the clade of x is not in G*'.

*Proof: *Suppose otherwise that there is some *x*' *∈ V *(*G*') with the same clade as *x *(and hence *s*(*x*) = *s*(*x*')). Let *r*'*_a,b _*= lca*_G' _*(*a, b*), which should be a speciation. Since *b *was not in the clade of *x*, it cannot be in the clade of *x*' either, implying that *r*'_a,b _is an ancestor of *x*'. Also, since *s*(*x*') = *s*(*x*) and *x *is above *h_a,b _*in *G*, we have that *s*(*x*') is *s_a,b _*or one of its ancestors (otherwise we would have picked *x *to be *h_a,b_*). But *r*' has *x*' in one of its subtrees, and *b *in the other, implying that *r*'_a,b _is a duplication: contradiction.    □

We now have a way to identify a set of clades that cannot be in *G_P_*. For any (*a, b*) ∈ *P_f_*, denote by *H_a,b _*the set of ancestors of *h_a,b _*that are descendants of *r_a,b_*. If *G_P _*satisfies the set *P_f_*, *G_P _*cannot contain any clade from the set $H=\cup_{\left(a,b\right)\in P_{f}}H_{a,b}$. It follows that a minimum of *|H| *clades of *G *are missing in *G_P_*. We claim that a solution *G_P _*to the GOC problem is obtained by modifying exactly *c*(*G, G_P_*) = *|H| *clades.

**Theorem 1 ***Let G_P _be a solution to the GOC problem. Then RF *(*G, G_P_*) = 2*|H|*.

In what follows, we give a constructive proof of Theorem 1 by describing an algorithm for solving the GOC problem.

### An algorithm for the GOC problem

Call *V *(*G*)*\H *the set of *preservable nodes *of *G *(those that we hope to preserve). For example in Figure 2(1), $H=H_{a_{1},c_{2}}\cup H_{c_{2},a_{1}}\cup H_{a_{1},c_{1}}\cup H_{c_{1},a_{1}}\cup H_{a_{1},b_{2}}\cup H_{b_{2},a_{1}}=\left\{e_{1}\right\}\cup \left\{e_{2}\right\}\cup \emptyset \cup \emptyset \cup \left\{d_{1}\right\}\cup \emptyset =\left\{e_{1},e_{2},d_{1}\right\}$.The nodes of *H *are represented by red crosses, while the preservable nodes are circled in green. Notice that the root *r *of *G *is preservable, since any solution *G_P _*to the GOC problem should share the same leaf-set as *G*. Consider the set  G of subtrees of *G *rooted on the *highest preservable descendants *of *r*, i.e. preservable nodes for which *r *is the unique preservable ancestor. Observe that since any leaf of *G *is preservable, we have $\cup_{G_{x}\in \;G}l\left(G_{x}\right)=l\left(G\right)$. If, for some (*g*_1_*, g*_2_) ∈ *P*, *g*_1 _and *g*_2 _are scattered across two subtrees of *G*, we call these subtrees *required orthologous subtrees *(or simply *required orthologs *when the context is clear as to whether we are comparing genes or subtrees). For example in the tree *G *of Figure 2(1), *G *is the set of subtrees rooted at *d*_2_, *c*_1_, *b*_3 _and *c*_2 _(the last four restricted to a single leaf), and the subtrees rooted at *d*_2 _and *c*_1 _are required orthologs, as well as those rooted at *d*_2 _and *c*_2_. However, connecting two subtrees under a speciation might not always be feasible. A definition of *possible orthologs *follows.

**Definition 1 (Possible orthologs) ***Two subtrees G*_1_*, G*_2 _∈  G*rooted at x*_1_*, x*_2 _*respectively are *possible orthologs *if and only if s*(*x*_1_) *and s*(*x*_2_) *are unrelated, i.e. neither is an ancestor of the other in S*.

The following lemma ensures that the roots of required orthologous subtrees can actually be joined under a common parent which is a speciation.

**Lemma 2 ***Let G*_1_*, G*_2 _∈  G*be required orthologs. Then G*_1 _*and G*_2 _*are possible orthologs*.

*Proof: *Let *x*_1_*, x*_2 _be the roots of *G*_1_*, G*_2 _respectively, and let (*g*_1_*, g*_2_) ∈ *P *such that *g*_1 _∈ *l*(*G*_1_) and *g*_2 _∈ *l*(*G*_2_). Let *s_ℓ_, s_r _*be the left and right children of $s_{g_{1},g_{2}}$, and denote by *S_ℓ _*and *S_r _*the subtrees of *S *rooted at *s_ℓ _*and *s_r _*respectively. Suppose without loss of generality that *s*(*g*_1_) is in *l*(*S_ℓ_*) and *s*(*g*_2_) is in *l*(*S_r_*). Since *x*_1 _is preservable and on the path between *g*_1 _and $r_{g_{1},g_{2}}$, we have $x_{1}\notin H_{g_{1},g_{2}}$ and thus *s*(*x*_1_) ∈ *V *(*S_ℓ_*). Similarly, *s*(*x*_2_) ∈ *V *(*S_r_*). Therefore *s*(*x*_1_) and *s*(*x*_2_) are unrelated and possible orthologs.

The problem, formally defined in the sequel as the *maximum orthology tree*, consists in joining all trees of  G into a single tree *G*' in a way ensuring that each pair of possible orthologs is joined under a speciation. More precisely, for some possible orthologs $G_{1},G_{2}\in \;G$ rooted at nodes *x*_1_*, x*_2_, we get that lca_*G*'_(*x*1*, x*_2_) is a speciation, with *G*_1_*, G*_2 _being unchanged.

We begin by giving an overview of the whole algorithm.

Algorithm Outline:

1. Compute the set $H=\cup_{\left(a,b\right)\;\in \;P_{f}}H_{a,b}$ of internal nodes of *G *corresponding to clades that cannot be in *G_P_*;

2. Compute the set  G of subtrees rooted at the highest preservable descendants of the root of *G*. If  G is empty, return *G *and terminate;

3. Construct a tree *G*' by joining all trees of  G in a way ensuring that possible orthologs are joined under speciation. We call *G*' the *maximum orthology tree *for  G;

4. For every tree $G_{x}\in \;G$, construct *G_x,P _*by recursively repeating Steps 2 to 4 with *G *being *G_x_*, and replace the *G_x _*subtree of *G*' by *G_x,P_*.

The tree obtained corresponds to the corrected tree *G_P _*we want. Running this algorithm on the *G *tree of Figure 2 yields the corrected tree *G_P_*. This algorithm terminates, since we eventually reach all the leaves of *G*, which correspond to terminal cases in the recursion. Implementing step 1 is straightforward, while step 2 can be done by performing a depth-first search from the root, in which upon visiting a preservable node, we add it to  G and continue the search without visiting its children. Step 3 is the purpose of the next section, so assume for now that it can be performed correctly as stated. This algorithm can be implemented to run in *O*(*|P | × |V *(*G*)*|*) steps in the worst case, the main bottleneck being the computation of *H*. The algorithm correctness follows from the two lemmas below.

**Lemma 3 ***Any preservable node x of G is *preserved *in G_P_, meaning that the clade of G rooted at x is a clade of G_P_*.

*Proof: *Let *x *be a preservable node of *G *and *G_x _*be the subtree rooted at *x*. It is not hard to see that eventually, steps 2-4 will be run on *G_x _*and return a tree *G_x,P_*, which will itself be a subtree of the final corrected tree *G_P_*. As the algorithm only moves and reconnects subtrees of *G_x_*, we have that *l*(*G_x_*) = *l*(*G_x,P_*). Since *G_x,P _*is a subtree of *G_P_*, it follows that the clade of *x *is preserved in *G_P_*.

**Lemma 4 ***Let *(*g*_1_*, g*_2_) ∈ *P. Then g*_1 _*and g*_2 _*are orthologs in G_P_*.

*Proof: *Denote by *G_v _*the subtree rooted at *v*, for some *v *∈ *V *(*G*). Let *x *be a preservable node and *G_x,P _*be the subtree produced after running steps 2-4 on *G_x_*. Let *D *be the set of highest preservable descendants of *x*. We say that a gene pair (*g*_1_*,g*_2_) is *contained *in *G_x _*if *g*_1_*, g*_2 _∈ *l*(*G_x_*). We use induction on the height of the tree to show that all gene pairs in *P *that are contained in *G_x _*are orthologous in *G_x,P _*(which proves the lemma since *x *can be the root). This is trivially true for leaves as they are preservable and contain no gene pairs. We thus suppose by induction that for any *d *∈ *D*, gene pairs in *P *that are contained in *G_d _*are orthologous in *G_d,P_*. Let (*g*_1_*, g*_2_) ∈ *P *such that (*g*_1_*, g*_2_) is contained in *G_x_*, but there is no *d *∈ *D *such that *G_d _*contains (*g*_1_*, g*_2_). What is left to prove is that *g*_1 _and *g*_2 _are orthologous in *G_x,P_*.

We first observe that *g*_1_*, g*_2 _belong to two different subtrees $G_{d_{1}},G_{d_{2}}$, where *d*_1_*, d*_2 _∈ *D*. Otherwise $G_{d_{1}}=G_{d_{2}}$, implying that (*g*_1_*, g*_2_) is contained in $G_{d_{1}}$ and we are done. Therefore, $G_{d_{1}}$,$G_{d_{2}}$*are required orthologs, and hence possible orthologs. Since we may assume that G*_d1_ and *G*_d2_ are joined under a speciation in *G*_*x,P*_, we get that $\text{lc}{\text{a}}_{G_{x,P}}\left(g_{1},g_{2}\right)$ is a speciation. The result follows from observing that *G*_*x,P *_is a subtree of *G*_*P*_.

### Maximum orthology tree

We now describe a solution to the maximum orthology tree problem. Formally, given a set of *k *possible orthologous subtrees of *G *rooted on a set of nodes *X *= {*x*_1_, . . . *, x_k _*}, the problem is to construct a tree *F *with *l*(*F*) = *X*, such that for each pair *x_i_, x_j _*∈ *X *that correspond to roots of possible orthologs, *x_i _*and *x_j _*are orthologous in *F*.

Roughly speaking, the algorithm proceeds as follows: start with *F*_0 _being a copy of *S*. Iterate over *i *from 1 to *k*, at each step constructing *F_i _*by grafting *x_i _*on *F*_*i-*1 _right above the node *v *∈ *V *(*F*_0_) such that *s*(*v*) = *s*(*x_i_*). Proceeding this way, we show in Lemma 5 that nodes of *V *(*F*_0_) are ensured to remain speciation nodes all over the procedure, and in lemma 6 that the lowest common ancestor of two possible orthologs belongs to *V *(*F*_0_), leading to corollary 1 stating that possible orthologs are in fact orthologous in the output tree. Finally remove the leaves artificially introduced by *F*_0 _and *standardize *the tree, which means

• remove all nodes with no descendant labeled with extant genes;

• contract non-root degree 2 nodes, then contract the root if it is of degree one.

**Figure 3:**
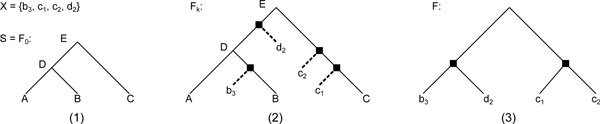
**The Max Orthology problem**. An instance of the *max orthology problem*, with *X *being the highest preservable descendants of the root of *G *in figure 2. (1) The starting tree *F*_0_, which is a copy of *S*. (2) The *F_k _*tree, which depicts the tree obtained after grafting every node of *X*. (3) The final tree *F*, obtained by removing the leaves initially in *F*_0 _and standardizing.

Starting with *F*_0 _being a copy of *S *is a step that might be omitted, but the set of nodes *V *(*F*_0_) serves as a skeleton around which we graft our *x_i_*'s, making it both easily implementable and provable. Figure 3 shows how the algorithm proceeds on the set of highest preservable descendants of the root of the tree *G *in Figure 2(1).

**Algorithm 1 ***findMaxOrthology*(*S, X *= {*x*_1_, ... *, x_k_*})

   *F*_0 _*← *A copy of *S*

   *V*_0 _*← V *(*F*_0_)

   *L ← l*(*F*_0_)

   **for ***i *= 1 *→ k ***do**

      Find the unique node *v *∈ *V*_0 _such that *s*(*v*) = *s*(*x_i_*)

      *F_i _← *a copy of *F_i - 1 _*on which we graft *x_i _*on the edge linking *v *to its parent node (or if *v *is the root of *F_i - 1_*, create a new root with children *v *and *x_i_*)

   **end for**

   *F ←F_k _*on which we remove *L *and stardardize

**Lemma 5 ***If r *∈ *V *(*F*_0_) ∩ *V *(*F*)*, then r is a speciation*.

*Proof: *Since *F*_0 _is a copy of *S*, all nodes of *V *(*F*_0_) are initially speciation nodes. We show that each grafting operation does not change the event corresponding to these nodes. Say that at iteration *i*, we graft *x_i _*on the edge linking *v *to its parent node *p*. We first observe that the only nodes that can be transformed from speciation in *F*_*i-1*_to duplication in *F_i _*are on the path from *p *to the root of *F*_*i-1*_. Suppose without loss of generality that *v *is the left child of *p *in *F*_*i-1*_, and let *w *be the newly created node between *p *and *v *in *F_i_*. Thus *w *has children *xi *and *v*, and since *s*(*x_i_*) = *s*(*v*), we get that *s*(*w*) = *s*(*v*). It follows that if *p *was a speciation in *F_i-1_*, it remains a speciation in *F_i_*. Moreover, this implies that *s*(*p*) is left unchanged in *F_i_*, implying in turn that any ancestor of *p *cannot change from speciation to duplication. Therefore, no grafting operation can affect speciation of any vertex in *V *(*F*_*i-1*_). Finally, we note that removing leaves or deleting degree two nodes in *F *also cannot affect speciation nodes.

**Lemma 6 ***Let x_i_, x_j _*∈ *X be the roots of possible orthologous subtrees. Then*, lca*_F _*(*x_i_, x_j_*) ∈ *V *(*F*_0_).

*Proof: *First recall that if *x_i_,x_j _*are the roots of possible ortholog subtrees, then there is some *s *∈ *V *(*S*) such that *s*(*x_i_*) and *s*(*x_j _*) are in the left and right subtrees of *s*, respectively. Now, let *r *be the unique node in *V *(*F*_0_) such that *s*(*r*) = *s*, and let *v_i_, v_j _*∈ *V *(*F*_0_) such that *s*(*v_i_*) = *s*(*x_i_*) and *s*(*v_j_*) = *s*(*x_j_*). It is clear that in *F*_0_, lca(*v_i_,v_j_*) = *r*. This also holds for any *F_i _*by observing that grafting nodes cannot change the lca relationship. Since *x_i _*is grafted on some edge between *v_i _*and *r*, and *x_j _*between *v_j _*and *r*, it follows that lca(*x_i_, x_j _*) = *r *∈ *V *(*F*_0_).

**Corollary 1 ***Let x_i_, x_j _*∈ *X be the roots of possible orthologs. Then they are orthologous in F*.

## The Clade Orthology Correction Problem

We prove several results characterizing the solutions to the COC problem. Let *C *be a set of clades that has to be satisfied. For a clade *c *∈ *C*, we denote by *s*(*c*) the value of *s*(*r*(*c*)) where *r*(*c*) is the root of *c*, and by *E*(*c*) the value of *E*(*r*(*c*)) that we call *the label of c*.

First, unlike in the GOC problem, a solution to the COC problem does not always exist. Indeed, it is possible that no gene tree has all clades in *C *labeled by speciations. We give a necessary and sufficient condition for the existence of a solution. The following lemma is obvious from the definition of reconciliation, and will be used in several proofs.

**Lemma 7 ***For a reconciled gene tree G, if a node x is an ancestor of a node y and s*(*x*) = *s*(*y*) *then E*(*x*) = *duplication*.

**Theorem 2 ***There is a solution to the COC problem if and only if for every clade c *∈ *C, s*(*c*) *is not a leaf of S, and if for every pair c*_1_*, c*_2 _∈ *C, either c*_1 _*and c*_2 _*are disjoint sets of leaves, or s*(*c*_1_) ≠ *s*(*c*_2_).

The necessity of these conditions directly follow from Lemma 7, since *s*(*c*_1_)*, s*(*c*_2_) and the ancestry relationship between *c*_1 _and *c*_2 _remain unchanged in a solution. Their sufficiency will be constructively demonstrated in the sequel. Suppose that the conditions are satisfied. We give a way of finding all optimal solutions according to the RF distance, followed by two ways of finding an optimal one optimizing other criteria in addition.

Given a duplication node *x *of *G*, *pushing x by multifurcation *means applying the following procedure:

• Let *s *= *s*(*x*), and *A *and *B *be the two children of *s *in *S*.

• Let *T^A ^*be the set of maximal subtrees of the subtree of *G *rooted at *x*, such that all their leaves *l *verify that *s*(*l*) is a descendant of *A *(including *A *itself). Let *G^A^*[*x*] be the multifurcated tree obtained by joining all roots of trees in *T^A ^*under a common root.

• Let symmetrically *T^B ^*be the set of maximal subtrees of the subtree of *G *rooted at *x*, such that all their leaves *l *verify that *s*(*l*) is a descendant of *B *(including *B *itself). Let *G^B^*[*x*] be the multifurcated tree obtained by joining all roots of trees in *T^B ^*under a common root.

• Let *G*' be obtained from *G *by replacing the clade rooted at *x *by a new subtree, obtained by joining *G^A^*[*x*] and *G^B^*[*x*] under a common root.

This rearrangement is described in [16] and applied to dubious duplications as a preprocessing step for ancestral genome reconstruction.

A *binary resolution G_b _*of a multifurcated tree *G *is a binary tree in which all the clades of *G *are in *G^b^*.

**Theorem 3 ***If there is a solution to the COC problem, then a binary gene tree is an optimal solution if and only if it is a binary resolution of the multifurcated tree obtained by pushing the roots of the elements of C by multifurcation (in any order)*.

*Proof: *It is clear that a binary resolution is a solution, provided that the conditions for the existence of a solution are satisfied. Indeed any clade is preserved through pushing a duplication node, so this operation can be done for all clades in *C *independently. This proves the converse part of Theorem 2.

Then it is an optimal solution because by Lemma 7, no clade *x *which is a descendant of the pushed clade *c *such that *s*(*c*) = *s*(*x*) may be conserved if we want *c *to be a speciation node. And by construction all clades such that *s*(*c*) ≠ *s*(*x*) are preserved by this operation.

Binary resolutions which minimize the number of duplications and losses are studied by [22] and may be applied to provide *bona fide *phylogenies. We describe an alternative maximizing the number of common triplets. A *triplet *in a tree *G *is a set of three leaves ((*a, b*)*, c*) of *G*, such that the LCA of the three is strictly more ancient than the LCA of the first two.

**Figure 4:**
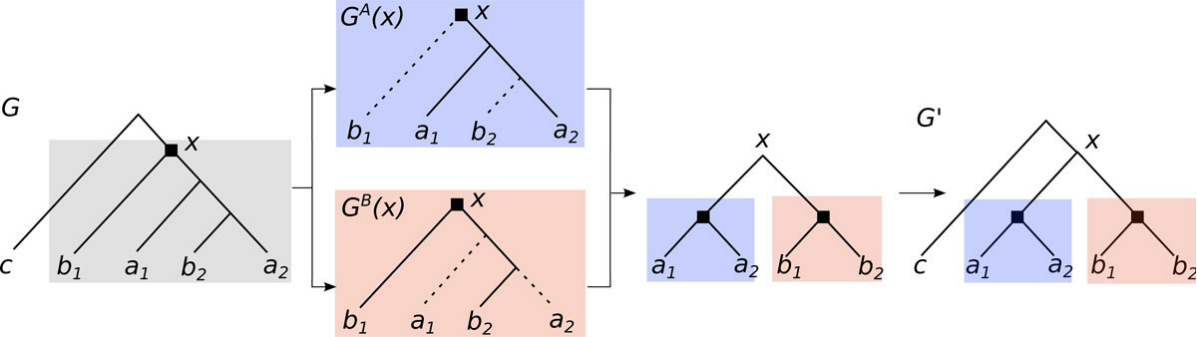
**COC Procedure**. An instance of the COC problem. In the gene tree G, where s(*a*_1_) = s(*a*_2_) = *A*, s(*b*_1_) = s(*b*_2_) = *B *and s(*c*) = *C*, extract and copy the subtree rooted at × to get the subtrees *G^A^*(*x*) and *G^B^*(*x*). Remove *b*_1 _and *b*_2 _from *G^A^*(*x*) and *a*_1 _and *a*_2 _from *G^B^*(*x*). Join *G^A^*(*x*) and *G^B^*(*x*) under a common root and replace G(x) by the new subtree in the gene tree G'.

Given a species tree *S*, a reconciled gene tree *G *and one of its duplication nodes *x*, *pushing x by tree duplication *means applying the following procedure, illustrated in Figure 4:

• Let *s *= *s*(*x*), and *A *and *B *be the two children of *s *in *S*.

• Let *G^A^*[*x*] be a tree obtained from the subtree of *G *rooted at *x*, by deleting all leaves *l *with *s*(*l*) being a descendant of *A*, and standardizing it, which as in the previous sections, means

**- **removing all nodes with no descendant labeled with extant genes;

**- **contracting non-root degree 2 nodes, then contracting the root if it is of degree one.

• Let symmetrically *G^B^*[*x*] be a tree obtained from the subtree of *G *rooted at *x*, by deleting all leaves *l *with *s*(*l*) being a descendant of *B*, and standardizing it.

• Let *G*' be obtained from *G *by replacing the clade rooted at *x *by a new subtree, obtained by joining *G^A^*[*x*] and *G^B^*[*x*] under a common root.

Note that if a clade *y *is disjoint from *x *or assigned to a different species, then pushing *x *by tree duplication does not affect the subtree rooted at *y*. In consequence, pushing several clades by tree duplications in any order gives a unique solution if the clades satisfy the properties of Lemma 2.

**Theorem 4 ***If there is a solution to the Clade Orthology Correction problem, the gene tree obtained by successively pushing the roots of the elements of C by tree duplication (in any order) is an optimal solution. Among all optimal solutions, it maximizes the number of common triplets with G*.

*Proof: *As already noticed pushing a duplication by multifurcation preserves all clades assigned to species which are different from the species assigned to the pushed node. So it is an optimal solution.

Now we have to prove that none of the triplets that are in *G *but not in *G*' can be preserved in any other optimal solution. For this we characterize the triplets that can be preserved. For a triplet ((*a, b*)*, c*) of *G*, let *T*_((*a,b*)*,c*) _be the rooted phylogeny with three leaves and two internal nodes containing the triplet. If the leaves *a, b, c *are in the pushed clade *x*, then the triplet can be preserved only if in the reconciliation of *T*_((*a,b*)*,c*)_, the lowest internal node is not mapped to *s*(*x*). Otherwise by Lemma 7, the root node of the triplet cannot be a speciation.

Let ((*a, b*)*, c*) be a triplet such that in the reconciliation of *T*_((*a,b*)*,c*)_, the lowest internal node is not mapped to *s*(*x*). This triplet is entirely included in *G*^1^[*x*] or *G*^2^[*x*]. So it is preserved. In consequence all triplets possibly preserved are indeed preserved by the operation, showing the optimality of the procedure reguarding the number of common triplets.

Now if there is no solution to the Clade Orthology problem, we advice to push duplication nodes in *C *starting from the highest ones, without having formalized why we find this solution adequate.

## Fish gene trees

Using synteny as evidence of orthology, we wanted to test the ability of our algorithm designed for the GOC problem to correct gene trees. To this end, we considered the four fish genomes *Gasterosteus aculeatus *(Stickleback), *Oryzias latipes *(Medaka), *Tetraodon nigroviridis*, and *Danio rerio *(Zebrafish) with human and mouse as outgroups. We used the *Ensembl Genome Browser *to collect all available gene trees, and filtered each tree to preserve only genes from the taxa of interest. We then reconciled the trees with the known species trees, and identified duplication and speciation nodes. Following our methodology in [11], a region surrounding a gene is defined as the substring containing the gene and both its left and right adjacencies, and two regions are considered syntenic if they contain homologous genes in the same order. We observed in [11] that more than 22% of the 6241 collected gene trees contain at least one false paralogy, that is a pair of genes required from synteny to be orthologous, but the LCA of the corresponding leaves being a duplication rather than a speciation node.

For 1000 of the trees containing at least one false paralogy, we applied the correction procedure previously described, and retrieved the gene family alignment from Ensembl. With PhyML [23], we computed the likelihood of the initial and corrected tree, given the alignment. These two likelihood values were compared with Consel [24]. For only 17.7% of the trees, the correction was rejected by the AU test. In other words, the correction algorithm is valid for a vast majority (82.3%) of the tested trees. Moreover, the likelihood of the corrected tree is higher than the original for 44.4% of the trees. Interestingly, 14.8% of the original Ensembl gene trees were rejected when compared to the corrected trees.

**Figure 5:**
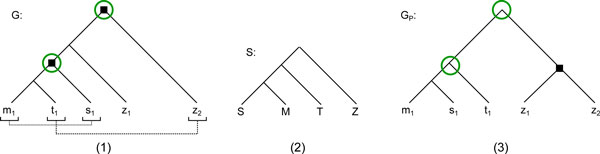
**An example of corrected fish tree**. The tree for the ZNF800 gene family before and after correction, restricted to the species Stickleback (S), Medaka (M), Tetraodon (T) and Zebrafish (Z). (1) The original gene tree *G *given by Ensembl, using the same notation as in figure 2 for duplications, preservable nodes and required orthologs. Gene region analysis gave us the required orthologs *P *= {(*m*_1_*, s*_1_), (*t*_1_*,d*_2_)}. (2) The species tree associated with the four species. (3) The gene tree given by our correction algorithm.

The correction of the gene tree for the *ZNF800 *gene family, which is related to transcriptional regulation, is given as an example in Figure 5. The corrected tree was highly favored by the AU Test, giving it a statistical support advantage with a p-value below 0.001. Furthermore, the non-apparent duplication of *G*, located at the root of the (*m*_1_*, t*_1_*, s*_1_) subtree, was eliminated, resulting in one less duplication in *G_P_*.

## Conclusion

We give two efficient algorithms for two new gene tree rearrangement problems, related to the correction of a gene tree according to some external information on orthology. The rearrangements are modifications that are as small as possible, given some distance criterion (namely the RF distance), but can be more significant according to other distances such as the usual NNI (nearest neighbor interchange) distance. We show that for fish genomes, the rearrangements we define can be efficient to explore statistically equivalent gene trees when sequence alignement is used to compute likelihood. As corrected trees satisfy synteny contraints, we can be confident enough that they describe the gene family evolution better.

Many algorithmic and theoretical problems remain open. For example, is there a similar way for handling paralogy constraints? What about having both orthology and paralogy constraints? It can be shown that there exist sets of constraints with both types that cannot be satisfied. What are the conditions for a set of orthology/paralogy constraints to be satisfiable?

These algorithms may be used in a global framework to contruct large gene tree sets which are arguably better than those found in standard databases. The implementation of such a framework is an on-going work.

## Competing interests

None

## Authors' contributions

ML, MS, KS, ET, NE modeled the problem, devised the algorithms and wrote the paper. ML implemented the software.

## Declarations

This work is funded by the Natural Sciences and Engineering Reserach Council of Canada (NSERC), Fonds de Recherche Nature et technologies of Quebec, Agence Nationale pour la Recherche and Ancestrome project ANR-10-BINF-01-01.

This article has been published as part of *BMC Bioinformatics *Volume 14 Supplement 15, 2013: Proceedings from the Eleventh Annual Research in Computational Molecular Biology (RECOMB) Satellite Workshop on Comparative Genomics. The full contents of the supplement are available online at http://www.biomedcentral.com/bmcbioinformatics/supplements/14/S15.
